# Construction of a circular RNA-based competing endogenous RNA network to screen biomarkers related to intervertebral disc degeneration

**DOI:** 10.1186/s12891-022-05579-0

**Published:** 2022-07-15

**Authors:** Bin Yu, Ziqi Zhu, Tao Hu, Jiawei Lu, Beiduo Shen, Tongde Wu, Kai Guo, Surendra Kumar Chaudhary, Hang Feng, Weidong Zhao, Desheng Wu

**Affiliations:** 1 Department of Spine Surgery, Shanghai East Hospital, School of Medicine, Tongji University, 150 Jimo Road, Shanghai, 200092 China; 2 Department of Surgery of Spine and Spinal Cord, Henan Provincial People’s Hospital, People’s Hospital of Zhengzhou University, People’s Hospital of Henan University, Zhengzhou, 450003 Henan China

**Keywords:** Intervertebral disc degeneration, Circular RNA, microRNA, mRNA, Competing endogenous RNA

## Abstract

**Background:**

Intervertebral disc degeneration (IDD) is a leading cause of disability with limited treatment strategies. A better understanding of the mechanism of IDD might enable less invasive and more targeted treatments. This study aimed to identify the circular RNA (circRNA)–microRNA (miRNA)–messenger RNA (mRNA) competing endogenous RNA (ceRNA) regulatory mechanisms in IDD.

**Methods:**

The GSE67567 microarray dataset was downloaded from the Gene Expression Omnibus database. After data preprocessing, differentially expressed circRNAs, miRNAs and mRNAs between IDD and controls were identified. A ceRNA network was constructed on the basis of the interaction between circRNAs and miRNAs, and miRNAs and mRNAs. Pathway enrichment analysis was performed on the mRNAs in the ceRNA network. Then, with ‘intervertebral disc degeneration’ as keywords, IDD-related Kyoto Encyclopedia of Genes and Genomes (KEGG) pathways were searched for in the Comparative Toxicogenomics Database.

**Results:**

A total of 105 differentially expressed circRNAs, 84 miRNAs and 967 mRNAs were identified. After analysis, 86 circRNA–miRNA, and 126 miRNA–mRNA regulatory relationship pairs were obtained to construct a ceRNA network. The mRNAs were enriched in six KEGG signalling pathways, and four were associated with IDD: the hsa04350: TGF-beta signalling pathway, hsa04068: FoxO signalling pathway, hsa05142: Chagas disease (American trypanosomiasis) and hsa04380: Osteoclast differentiation. An IDD-related ceRNA network was constructed involving four circRNAs, three miRNAs and 11 mRNAs. Auxiliary validation showed that the expression levels of miR-185-5p, miR-486-5p, *ACVR1B*, *FOXO1*, *SMAD2* and *TGFB1* were consistent in different databases.

**Conclusions:**

Our study identified some circRNA–miRNA–mRNA interaction axes potentially associated with the progression of IDD, viz*.*: circRNA_100086–miR-509-3p–MAPK1, circRNA_000200–miR-185-5p–TGFB1, circRNA_104308–miR-185-5p–TGFB1, circRNA_400090–miR-486-5p–FOXO1 and circRNA_400090–miR-486-5p–SMAD2.

**Supplementary Information:**

The online version contains supplementary material available at 10.1186/s12891-022-05579-0.

## Highlights

1. An IDD-related ceRNA network involving four circRNAs, three miRNAs and 11 mRNAs was constructed.

2. The expression levels of miR-185-5p, miR-486-5p, *FOXO1*, *SMAD2* and *TGFB1* were consistent in different databases.

3. Our study identified IDD-related circRNA–miRNA–mRNA interaction axes, including circRNA_100086–miR-509-3p–*MAPK1*.

**Supplementary Information:**

The online version contains supplementary material available at 10.1186/s12891-022-05579-0.

## Background

Intervertebral disc degeneration (IDD) is a multifactorial process characterised by increased extracellular matrix decomposition and decreased hydration due to abnormal matrix synthesis, loss of disc height and reduced ability to absorb load [1, 2]. IDD can lead to low back pain, a leading cause of disability that has huge impacts on the economy and life quality of patients worldwide [3, 4]. The aetiology of IDD is multifactorial, including ageing, certain injuries and diseases and genetic factors. Currently, the treatment for IDD is mainly limited to the use of steroidal or non-steroidal anti-inflammatory drugs to relieve symptoms and surgical intervention for late-stage IDD with severe neurological symptoms [5]. A better understanding of the mechanism of IDD would enable treatments that were less invasive and better targeted [5, 6].

Recently, new genetic tools have improved our understanding of the molecular basis of IDD, with genetic factors now known to be key contributors to its progression [7, 8]. Recent evidence has shown that noncoding RNAs, such as microRNAs (miRNAs), circular RNAs (circRNAs), and long noncoding RNAs (lncRNAs) are involved in several biological processes [9]. CircRNAs and lncRNAs can competitively bind to miRNAs through their miRNA response elements, thus, acting as competing endogenous RNAs (ceRNAs) to regulate the expression of miRNA–target messenger RNAs (mRNAs) [10]. ceRNA regulatory interactions have been reported to play important roles in the initiation and development of many diseases. For instance, Khan established circRNA-miRNA-mRNA regulatory axis in various cancers using state-of-the-art bioinformatics tools, such as hsa_circ_0051620| SLC1A5—hsa-miR-338-3p—CDH2 in lung cancer, hsa_circ_0074817| EBF1-hsa-miR-539-5p—CDK4 and SPAG5 in liver cancer [11]. Xiong et al. found that hsa_circRNA_100291 and hsa_circRNA_104515 may function as ceRNAs to exert critical roles in HCC [12]. Dong et al. uncovered six circRNAs (hsa_circ_0000390, hsa_circ_0000615, hsa_circ_0001438, hsa_circ_0002190, hsa_circ_0002449 and hsa_circ_0003120) that might function as ceRNA to play important roles in gastric cancer by establishing circRNA-miRNA-mRNA regulatory networks [13]. Bai et al. predicted that 3 circRNAs (hsa_circ_0029340, hsa_circ_0025135, hsa_circ_0039238) were ascertained as ceRNA to regulate the expression of 6 miRNAs (miR-1205, miR-657, miR-587, miR-637, miR-1278, miR-548p) in clear cell renal cell carcinoma [14]. Except for cancers, Sakshi et al. reviewed that circRNA-miRNA-mRNA interaction network that influences the gene expression in the progression of diabetes and its associated complications [15]. There are also studies establishing the ceRNA regulatory networks in IDD [16–20]. Besides, several ceRNA axis were confirmed by mechanistic study. For example, circARL15 plays a critical role in IDD by modulating miR-431-5p/DISC1 [21]. LncRNA HCG18 promotes IDD by sponging miR-146a-5p and regulating TRAF6 expression [22]. CircRNA_104670 plays a crucial role in IDD by functioning as a ceRNA [23]. CircRNA_001653 silencing promotes the growth of nucleus pulposus cells via the miR-486-3p/CEMIP axis in IDD [24]. Thus, we speculated that preventing or reversing IDD at the molecular level may have potential as a treatment. However, the ceRNA networks in IDD are far from being fully identified.

Hence, the present study aimed to further identify circRNA–miRNA–mRNA ceRNA regulatory mechanisms in IDD by establishing a ceRNA regulatory network on the basis of the circRNA, miRNA and mRNA profiling datasets in the Gene Expression Omnibus (GEO) database. Our results may deepen our understanding of the molecular mechanism of IDD.

## Methods

### Data grouping and preprocessing

The dataset GSE67567 [25–28] was downloaded from the NCBI GEO database. This dataset contains three subsets: GSE67566, GSE63492 and GSE56081. GSE67566 contains circRNA expression profiles (detection platform: Agilent); GSE63492 contains miRNA expression profiles (detection platform: Exiqon miRCURY LNA); GSE56081 contains mRNA–lncRNA expression profiles (detection platform: Agilent). Each of the datasets contains 10 nucleus pulposus samples derived from five normal (control) individuals and five patients with IDD.

For the GSE56081 data, the probe sequences were downloaded from the annotation platforms and aligned with the human genome using Clustal W (version 2.0) [29] to obtain the expression levels of mRNA and lncRNA.

### Differentially expressed RNA screening

According to their source, samples from the three datasets were divided into IDD and control groups. The RNA expression level differences (false discovery rate, and fold change (FC)) between IDD and control groups in each dataset were calculated using R3.4.1 Limma version 3.34.0, respectively [30]. FDR < 0.05 and |log_2_FC|> 1 were selected as the thresholds for significant differences for differentially expressed circRNAs, miRNAs and mRNAs. The expression values of circRNA, miRNA and mRNA in each sample were then extracted from the standardised expression profiles and used to perform Euclidean distance-based bidirectional hierarchical clustering [31] using pheatmap version 1.0.8 [32] in R3.4.1.

### Construction of circRNA and mRNA co-expression network

The Pearson correlation coefficient (PCC) [33] between the screened differentially expressed circRNAs and mRNAs was calculated using the cor.test function in R3.4.1. The co-expression network was built using Cytoscape 3.6.1 [34]. Afterwards, the mRNAs in the network were subjected to GO biological process and KEGG pathway enrichment analyses [35–37] using DAVID version 6.8 [38], with *p* < 0.05 selected as the significance threshold.

### Construction of ceRNA regulatory network

#### circRNA–mRNA network construction

We first downloaded the sequences of human circRNA from circBase [39] on 14^th^ May, 2021 (.fastq, 477 M) and then obtained the sequences of differentially expressed miRNA from the annotation platform of miRNA expression profile GSE63492. The binding relationship between target circRNAs and miRNAs was predicted through miRanda [40] (alignment parameters: Gap Open Penalty =  − 8, Gap Extend =  − 2, Score Threshold = 80%, and Energy Threshold =  − 20). Additionally, connection pairs with opposite difference directions were retained to construct the circRNA–miRNA network using Cytoscape 3.6.1.

#### miRNA–target network construction

Using the starBase version 2.0 database [41], the target genes of miRNAs that had connections with circRNAs were predicted. The starBase database comprehensively provided target gene prediction information from five databases: TargetScan, picTar, RNA22, PITA and miRanda. We selected the regulatory relationships that were included in at least one of the databases as the miRNA–target regulatory relationship pairs. Then, the differentially expressed mRNAs were matched to the target genes regulated by miRNAs, and the regulatory relationship pairs with opposite expression directions were retained to build the miRNA–mRNA regulatory network.

#### ceRNA regulatory network construction

By combining the circRNA–mRNA and miRNA–mRNA networks, a circRNA–miRNA–mRNA ceRNA network was constructed. Then, the mRNA contained in the network was enriched by reference to KEGG pathways using DAVID version 6.8 software, with *p* < 0.05 selected as the significance threshold.

### Construction of ceRNA regulatory network directly related to IDD

In the Comparative Toxicogenomics Database (CTD) 2022 update [42], the IDD-related KEGG pathways were searched using the string ‘intervertebral disc degeneration’ as a keyword. The obtained pathways were then compared with the pathways obtained in the previous step (ceRNA network) to identify the overlapped pathway manually. The overlapped mRNAs in the ceRNA network associated with these pathways were extracted, obtaining an IDD-related ceRNA regulatory network. The biological targets related to IDD were then identified.

### Auxiliary validation

The expression levels of the obtained biological targets were extracted from the GSE63492 and GSE56081 datasets. Two additional datasets were also downloaded from the NCBI GEO database for auxiliary validation of expression levels. GSE19943 is a miRNA expression profile, including six nucleus pulposus samples derived from three scoliosis (control) individuals and three patients with IDD. GSE34095 is an mRNA expression profile that includes six lumbar tissue samples from three younger patients with scoliosis (control) individuals and three IDD patients. The normalized (log2 transformed) expression data were downloaded and compared their expression levels between IDD group and control group using student’s *t* test. *P* < 0.05 was regarded as statistically significant level.

## Results

### A panel of differentially expressed genes was identified

**Fig. 1:**
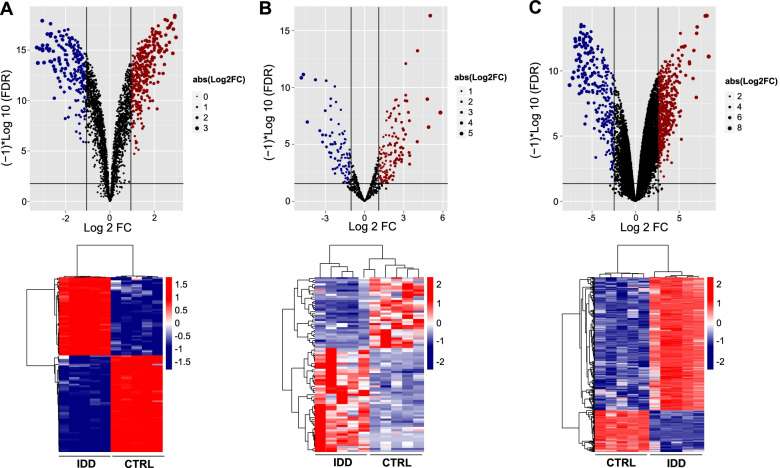
Identification of differentially expressed circRNAs, miRNAs and mRNAs between IDD group and control group. Volcano plots (up panel) and heat maps (low panel) based on significantly differentially expressed circRNA (**A**), miRNA (**B**) and mRNA (**C**). Blue and red dots indicate significantly downregulated and upregulated RNAs, respectively. The black horizontal line indicates FDR = 0.05, and the two vertical dashed lines indicate |log_2_FC|= 1

A total of 105 differentially expressed (47 upregulated and 58 downregulated) circRNAs, 84 (50 upregulated and 34 downregulated) miRNAs, and 967 (737 upregulated and 230 downregulated) mRNAs were identified. The volcano plots are shown in Fig. 1 (Up), and the bidirectional hierarchical clustering heat maps are shown in Fig. 1 (Down). As shown in the heat maps, the expression values of the three types of RNA were all fully capable of separating the samples of different types (IDD *vs* control) with distinct colours, indicating that the differentially expressed RNAs screened in the datasets had sample characteristics.

### A co-expression network was constructed

**Fig. 2:**
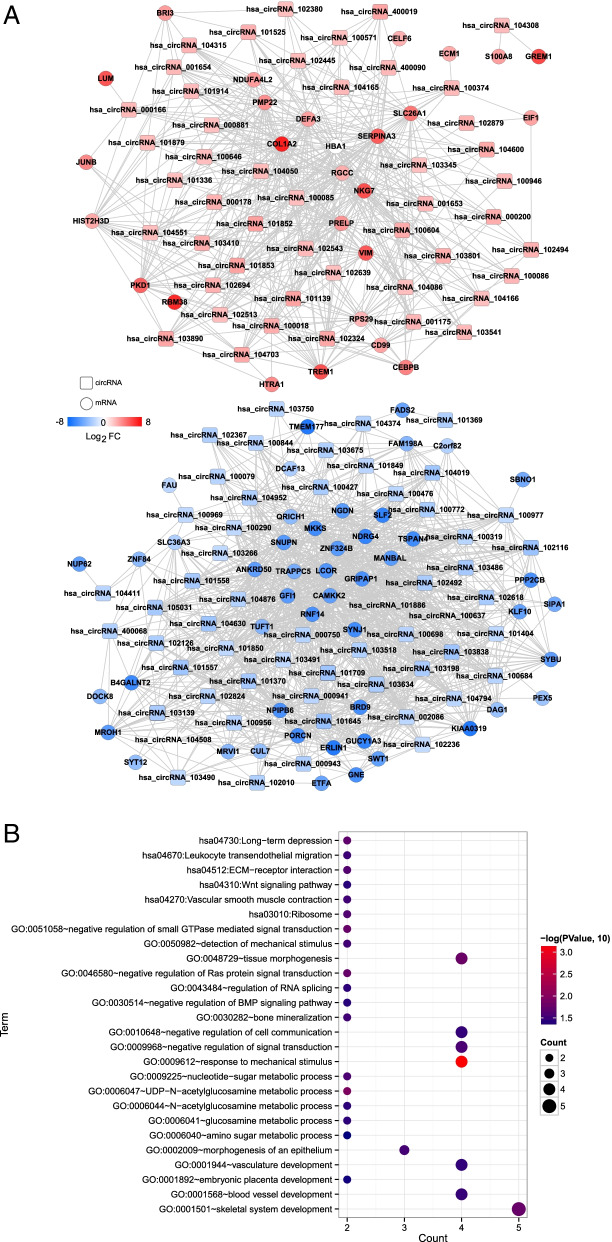
Construction and functional analysis of co-expression networks. **A** Co-expression network diagram of circRNAs and mRNAs with significant differences. The square represents the circRNAs, the circle represents the mRNAs and the colour change from blue to red represents the change in |Log_2_FC| from low to high. **B** Bubble diagram of GO biological process and KEGG signalling pathway of mRNAs in the co-expression network. The horizontal axis represents the number of genes, and the vertical axis represents the names of GO and KEGG signalling pathways. The size and colour of the node represent − log_10_(*p* value)

For the obtained differentially expressed circRNAs and mRNAs, expression levels were extracted from the GSE67567 dataset to calculate the PCC. Interaction pairs with PCC > 0.9 were retained, yielding 1599 co-expression interaction pairs. The co-expression network is shown in Fig. 2A. Functional enrichment analysis of the mRNAs in the network identified 20 biological processes (such as GO:0,009,612 ~ response to mechanical stimulus, GO:0,006,047 ~ UDP-N-acetylglucosamine metabolic process, GO:0,048,729 ~ tissue morphogenesis and GO:0,001,501 ~ skeletal system development) and six KEGG signalling pathways (hsa04730: Long-term depression, hsa04512: ECM-receptor interaction, hsa03010: Ribosome, hsa04270: Vascular smooth muscle contraction, hsa04670: Leukocyte transendothelial migration and hsa04310: Wnt signalling pathway) (Fig. 2B).

### A ceRNA network involving three miRNAs and four circRNAs was constructed

**Fig. 3:**
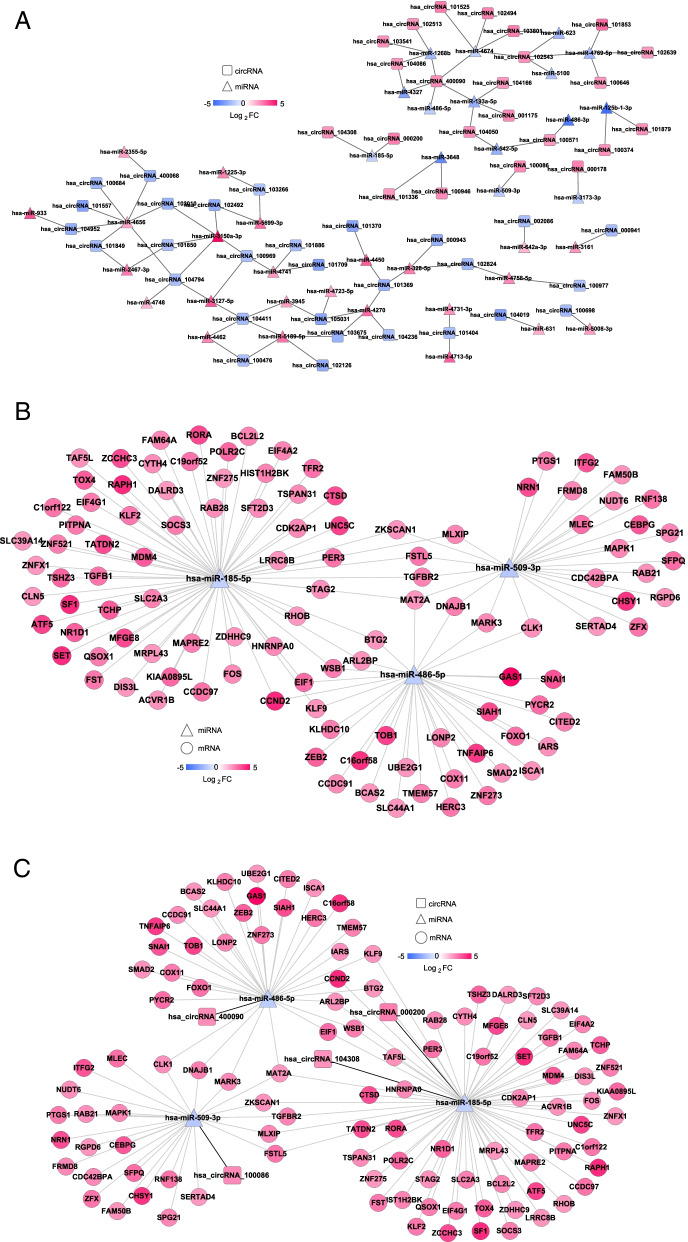
Construction of a ceRNA regulatory network. **A** Regulatory network diagram of the circRNAs and mRNAs with significant differences. **B** Regulatory network of miRNA–target. **C** ceRNA regulatory network. The square represents the circRNAs, the triangle represents the miRNAs, the circle represents the mRNAs and the colour change from blue to red represents the change in |Log_2_FC| from low to high

A total of 86 circRNA–miRNA pairs with opposite expression directions were identified, and a network was constructed (Fig. 3A). Additionally, 126 miRNA–mRNA regulatory relation pairs were obtained (Fig. 3B). Then, combining the circRNA–miRNA and miRNA–mRNA networks, a circRNA–miRNA–mRNA ceRNA network was constructed (Fig. 3C). There were three downregulated miRNAs (miR-486-5p, miR-509-3p and miR-185–5) and four upregulated circRNAs (circRNA_400090, circRNA_104308, circRNA_000200 and circRNA_100086) in the network.

### The ceRNA regulatory network directly related to IDD was established and validated in independent datasets

**Fig. 4:**
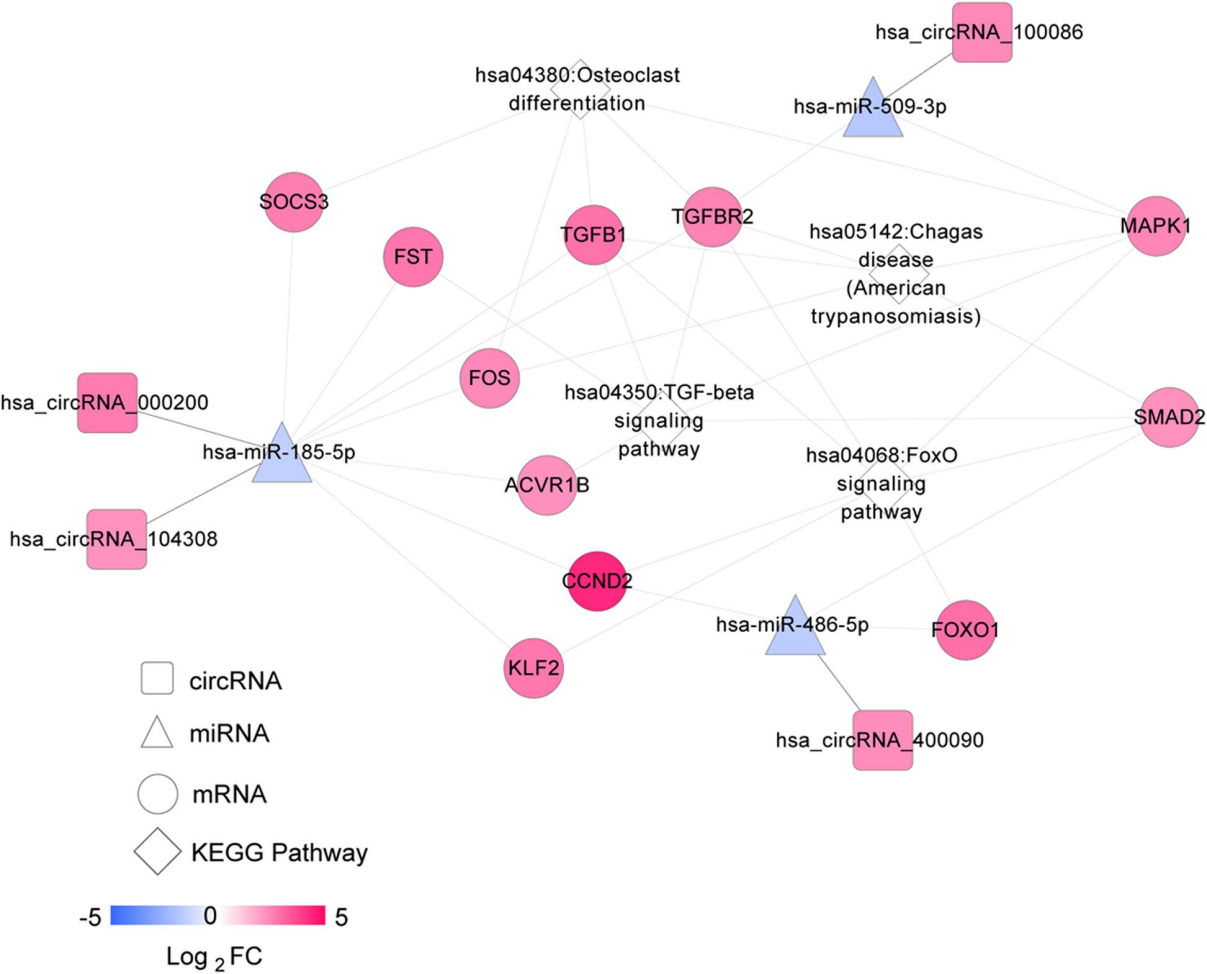
Intervertebral disc degeneration-related integrated ceRNA regulatory network. The rhombus represents the disease-related pathway, the square represents the circRNAs, the triangle represents the miRNAs, the circle represents the mRNAs and the colour change from blue to red represents the change in |Log_2_FC| from low to high

KEGG signalling pathway enrichment annotation was carried out on the target genes in the ceRNA regulatory network, and a total of 6 KEGG signalling pathways were obtained, viz*.*: hsa04350: TGF-beta signalling pathway, hsa04068: FoxO signalling pathway, hsa05142: Chagas disease (American trypanosomiasis), hsa04380: Osteoclast differentiation, hsa04520: Adherens junction and hsa04917: Prolactin signalling pathway. The CTD database was searched using the string ‘intervertebral disc degeneration’ as a keyword, and 68 KEGG signalling pathways directly related to IDD were identified. After comparison with KEGG pathways with significant enrichment of target mRNA in the comprehensive ceRNA network, four overlapping pathways were obtained, viz*.*: hsa04350: TGF-beta signalling pathway, hsa04068: FoxO signalling pathway, hsa05142: Chagas disease (American trypanosomiasis) and hsa04380: Osteoclast differentiation. The mRNAs in the ceRNA network involved in the four pathways was extracted to construct the IDD-related ceRNA network (Fig. 4). A total of four circRNAs (circRNA_100086, circRNA_000200, circRNA_104308 and circRNA_400090), three miRNAs (miR-509-3p, miR-185-5p and miR-486-5p) and 11 mRNAs (such as transforming growth factor beta 1 (*TGFB1*), mitogen-activated protein kinase 1 (*MAPK1*), forkhead box O1 (*FOXO1*), SMAD family member 2 (*SMAD2*) and activin A receptor type 1B (*ACVR1B*)) were involved in the network. For example, circRNA_000200 and circRNA_104308 could regulate the expression of suppressor of cytokine signalling 3 (*SOCS3)*, follistatin (*FST), TGFB1*, TGF beta receptor 2 (*TGFBR2),* Fos proto-oncogene, AP-1 transcription factor subunit *(FOS), AVR1B,* cyclin D2 *(CCND2)* and Kruppel like factor 2 (*KLF2)* by sponging miR-185-5p to participate in the pathways of hsa:04,380: Osteoclast differentiation, hsa:04,350: TGF-beta signalling pathway, hsa04068: FoxO signalling pathway or hsa05142: Chagas disease (American trypanosomiasis). CircRNA_400090 could regulate the expression of *FOXO1*, *CCND2* and *SMAD2* to participate the pathways of hsa04068: FoxO signalling pathway or hsa05142: Chagas disease (American trypanosomiasis). CircRNA_100086 could regulate the expression of *MAPK1* and *TGFBR2* by sponging miR-509-3p to participate in the pathways of hsa:04,350: TGF-beta signalling pathway, hsa04068: FoxO signalling pathway, hsa05142: Chagas disease (American trypanosomiasis) and hsa:04,380: Osteoclast differentiation. The binding sequences analysis of critical circRNAs, miRNAs and mRNAs were displayed in Additional files 1, 2 and 3.

**Fig. 5:**
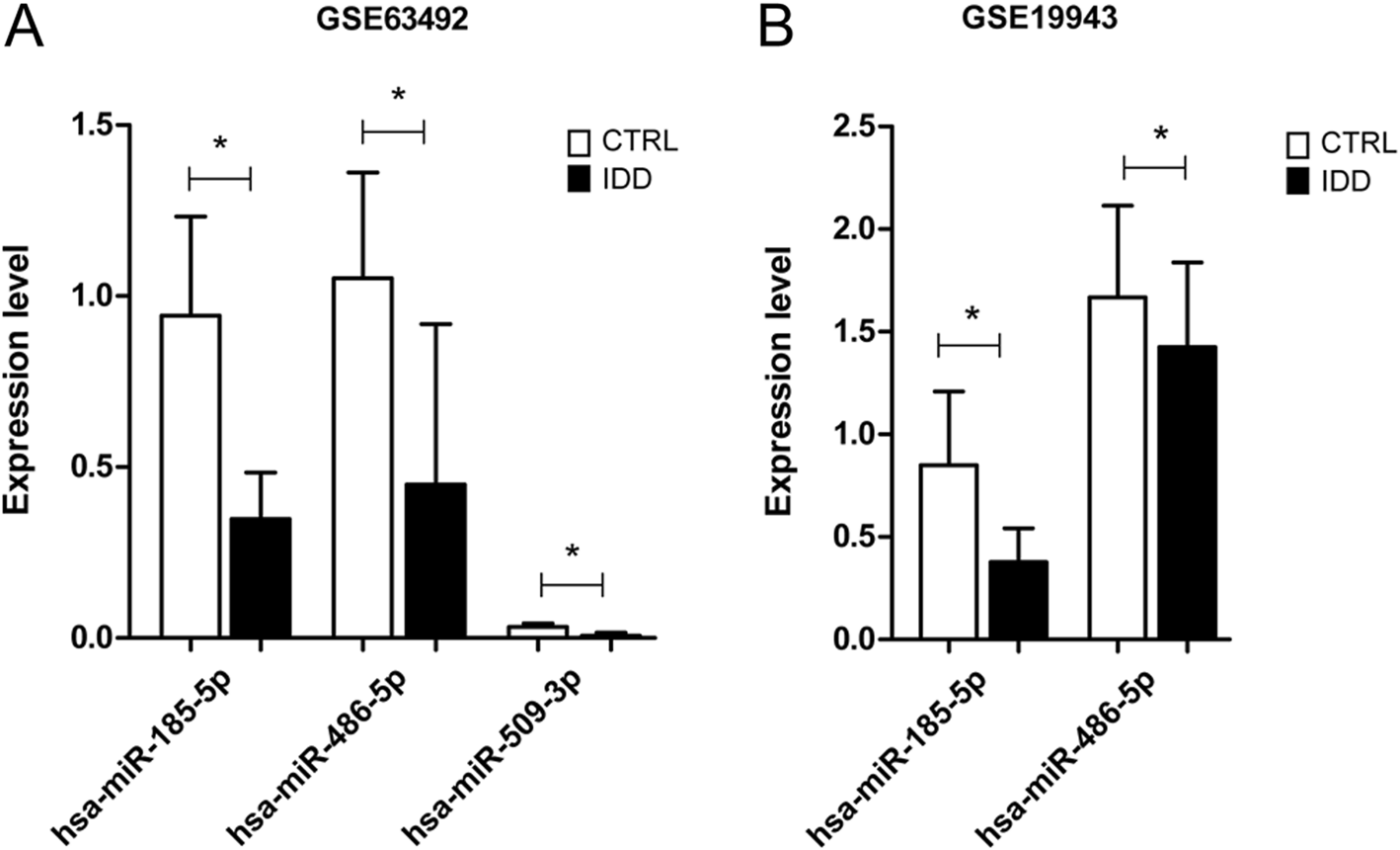
Validation of differential expression of miRNAs in the ceRNA network in independent dataset. Expression levels of three miRNAs in GSE63492 (**A**) and GSE19943 (**B**). The difference of expression level (log2 transformed expression) was compared between control (CTRL) group and IDD group by student’s t test. * represents *P* < 0.05

**Fig. 6:**
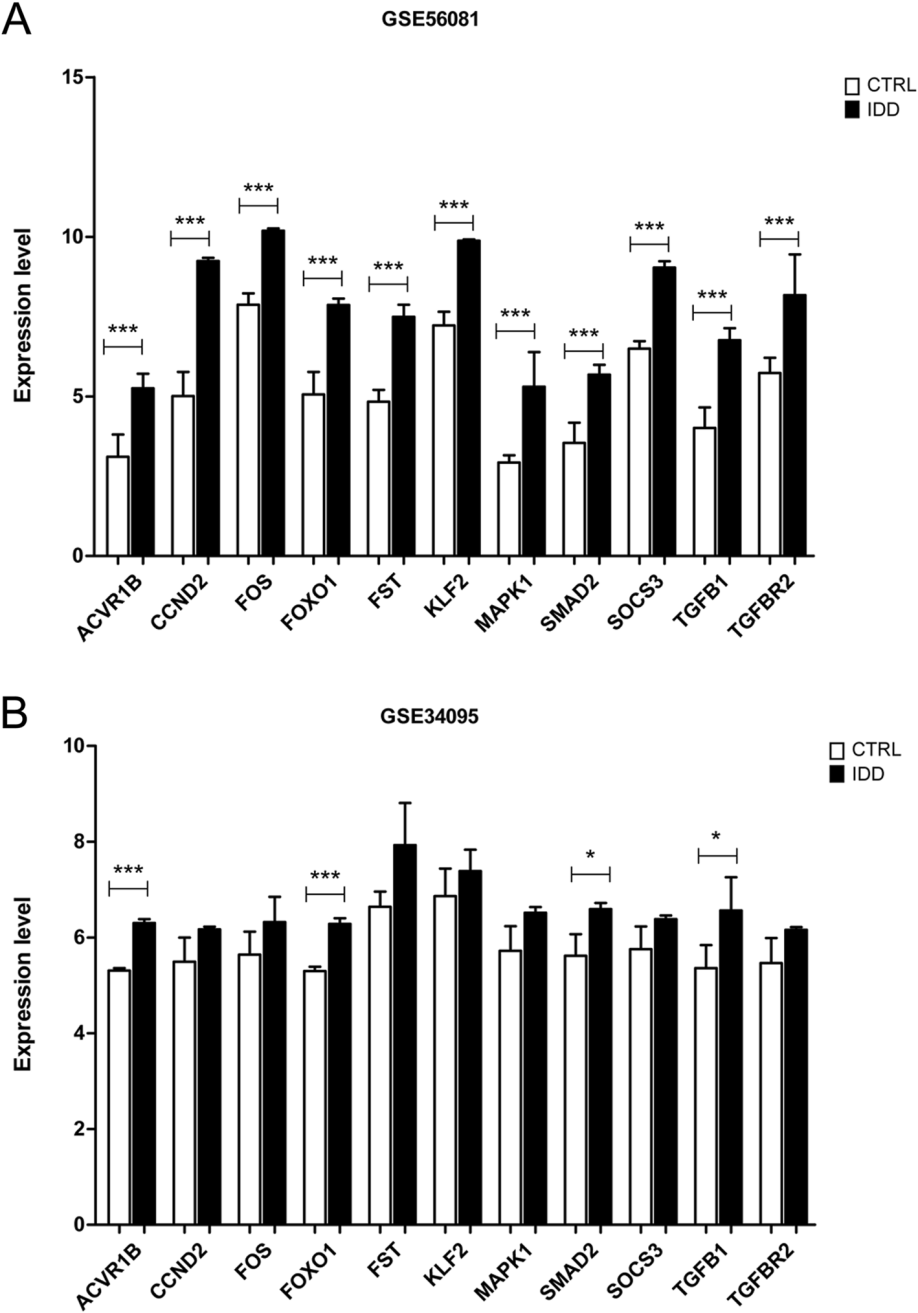
Validation of differential expression of mRNAs in the ceRNA network in independent dataset. Expression levels of 11 mRNAs in GSE56081 (**A**) and GSE34095 (**B**). The difference of expression level (log2 transformed expression) was compared between control (CTRL, patients with scoliosis) group and IDD group by student’s t test. * represents *P* < 0.05 and *** represents *P* < 0.001

In order to validate our results in independent datasets, we searched suitable datasets from GEO databases and obtained GSE19943 and GSE34095 for validating the expression of miRNAs and mRNAs in the ceRNA regulatory network, respectively. Unfortunately, there was no suitable dataset for auxiliary validation of circRNAs in the network. GSE19943 contains the miRNA expression profiles of human nucleus pulposus cells derived from patients with IDD and those from patients with scoliosis as control. GSE34095 includes expression data of intervertebral disc tissues harvested from elderly and younger patients with IDD and scoliosis, respectively. The normalized (log2 transformed) miRNA and mRNA data in the control group and IDD group from the training datasets GSE63492 and GSE56081 were displayed in Figs. 5A and 6A. Then the expression levels of corresponding miRNAs and mRNAs were extracted from GSE19943 and GSE34095, respectively (Figs. 5B and 6B). For the miRNA expression validation, two miRNAs (miR-185-5p and miR-486-5p) were detected in the GSE19943 dataset, and their expression levels were significantly decreased in IDD samples compared with those in control (*P* < 0.05), which was consistent with that in the original data set GSE63492. Eleven mRNAs in the ceRNA regulatory network were all significantly upregulated in IDD samples, compared with those in the control samples in the training dataset GSE56081 (*P* < 0.001). These mRNAs were also upregulated in the IDD tissues of validation dataset compared with those in the control tissues, among which the differences in *ACVR1B*, *FOXO1*, *SMAD2* and *TGFB1* were significant (*P* < 0.05) in GSE34095.

## Discussion

In this study, an IDD-related ceRNA network was constructed involving four circRNAs, three miRNAs and 11 mRNAs. Auxiliary validation showed that the expression levels of miR-185-5p, miR-486-5p, *ACVR1B*, *FOXO1*, *SMAD2* and *TGFB1* were consistent in different databases.

Reportedly, TGF-β can promote the proliferation of nucleus pulposus cells and stimulate ECM synthesis [43]. Hence, TGF-β has the potential to inhibit IDD [44]. In this study, the hsa04350: TGF-beta signalling pathway was found to be associated with IDD. The genes enriched in this pathway, such as *MAPK1* and *TGFB1*, were involved in the ceRNA regulatory network. The MAPK pathway has been implicated in IDD [45]. Differentially expressed MAPK isoforms in nucleus pulposus cells can modulate macrophage polarisation in IDD [46]. In the present study, circRNA_100086 can function as a ceRNA for regulating miR-509-3p target MAPK1. To our knowledge, there have been no studies on the roles of circRNA_100086 and miR-509-3p in IDD. We speculate that the circRNA_100086–miR-509-3p–MAPK1 axis may play an important role in IDD.

*TGF-β* is a regulatory protein that plays a crucial role in inflammatory events, and *TGFB1* is the most abundantly expressed member of the TGF-β family [47]. In the intervertebral disc, inflammatory processes can lead to disc degeneration. Particularly, an increase in inflammatory mediators promotes matrix degradation, disc cell senescence and death and the recruitment of immune cells, all of which lead to impaired biomechanical function of the intervertebral disc [48, 49]. In this study, *TGFB1* was regulated by miR-185-5p that interacted with circRNA_000200 and circRNA_104308. miR-185-5p has been reported to be associated with cancer progression [50, 51], but there are no reports of its having a role in IDD. We speculate that circRNA_000200/circRNA_104308–miR-185-5p–*TGFB1* may be involved in the development of IDD via inflammatory processes.

The FoxO signalling pathway was also directly related to IDD. FoxO is an evolutionarily conserved family of transcription factors that play an important role in ageing and longevity [52]. Reportedly, the FoxO transcription factor family is essential for the maturation and maintenance of the intervertebral disc [53]. Alvarez-Garcia et al. [54] also reported that FoxO is necessary for the homeostasis of the intervertebral disc during ageing and that a deficiency promotes disc degeneration. In the present study, *FOXO1* and *SMAD2* were enriched in this pathway and involved in the ceRNAs of circRNA_400090–miR-486-5p–*FOXO1*/*SMAD2*.

A recent study reported that FOXO1 activation can promote the expression of inflammatory cytokines and extracellular matrix degradation in nucleus pulposus cells in IDD [55]. SMAD proteins mediate signals from receptor serine–threonine kinases of the TGF-β superfamily [56]. A previous study has shown that platelet-rich plasma can promote the regeneration of nucleus pulposus cells through the TGF-β1/SMAD signalling pathway, in which SMAD2/3 is phosphorylated by TGF-β1 to induce the re-differentiation process of nucleus pulposus cells [57]. Recently, Yang et al. [58] reported that the TGF-β1/SMAD2/3 pathway plays a key role in the ability of platelet-rich plasma to retard IDD. Taken together, we speculate that circRNA_400090 may be involved in IDD by sponging miR-486-5p and regulating the expression of *FOXO1* and *SMAD2*.

There are some limitations in the study. Though the ceRNA network was established by state-of-the-art bioinformatics tools, and the miRNAs and mRNAs in the ceRNA regulatory network was validated in independent datasets, auxiliary validation of circRNAs in the network was not performed because no suitable dataset was available. Second, further in vivo and in vitro experiments are warranted for confirmingd the ceRNA regulatory axis in future.

## Conclusion

Our study identified some circRNA–miRNA–mRNA interaction axes, including circRNA_100086–miR-509-3p–*MAPK1*, circRNA_000200–miR-185-5p–*TGFB1*, circRNA_104308–miR-185-5p–*TGFB1*, circRNA_400090–miR-486-5p–*FOXO1* and circRNA_400090–miR-486-5p–*SMAD2*, which may be associated with the progression of IDD and which may therefore serve as biomarkers of IDD. To confirm these regulatory relationships in IDD, further validation experiments are required.

## Supplementary Information

**Additional file 1.** The binding sequences analysis of circRNA_000200/ circRNA_104308-miR-185-5p-TGFß1.

**Additional file 2**. The binding sequences analysis of circRNA_100086-miR-509-3p-MAPK1.

**Additional file 3.** The binding sequences analysis of circRNA_400090-miR-486-5p-FOXO1/SMAD2. 

## Data Availability

The datasets analysed during the present study are available in the GEO repository [GSE67567, https://www.ncbi.nlm.nih.gov/geo/query/acc.cgi?acc=GSE67567], [GSE19943, https://www.ncbi.nlm.nih.gov/geo/query/acc.cgi?acc=GSE19943] and [GSE34095, https://www.ncbi.nlm.nih.gov/geo/query/acc.cgi?acc=GSE34095].

## Abbreviations

circRNA: Circular RNA
miRNA: MicroRNA
ceRNA: Competing endogenous RNA
IDD: Intervertebral disc degeneration
lncRNA: Long noncoding RNA
FDR: False discovery rate
FC: Fold change
PCC: Pearson correlation coefficient
CTD: Comparative Toxicogenomics Database
*TGFB1*: Transforming growth factor beta 1
*MAPK1*: Mitogen-activated protein kinase 1
*FOXO1*: Forkhead box O1
*SMAD2*: SMAD family member 2
*ACVR1B*: Activin A receptor type 1B
