# Supplementary figures and images for: Construction of a circular RNA-based competing endogenous RNA network to screen biomarkers related to intervertebral disc degeneration
Source: BMC Musculoskelet Disord. 2022 Jul 15;23:675. doi: 10.1186/s12891-022-05579-0 (PMC9284696; doi:10.1186/s12891-022-05579-0)

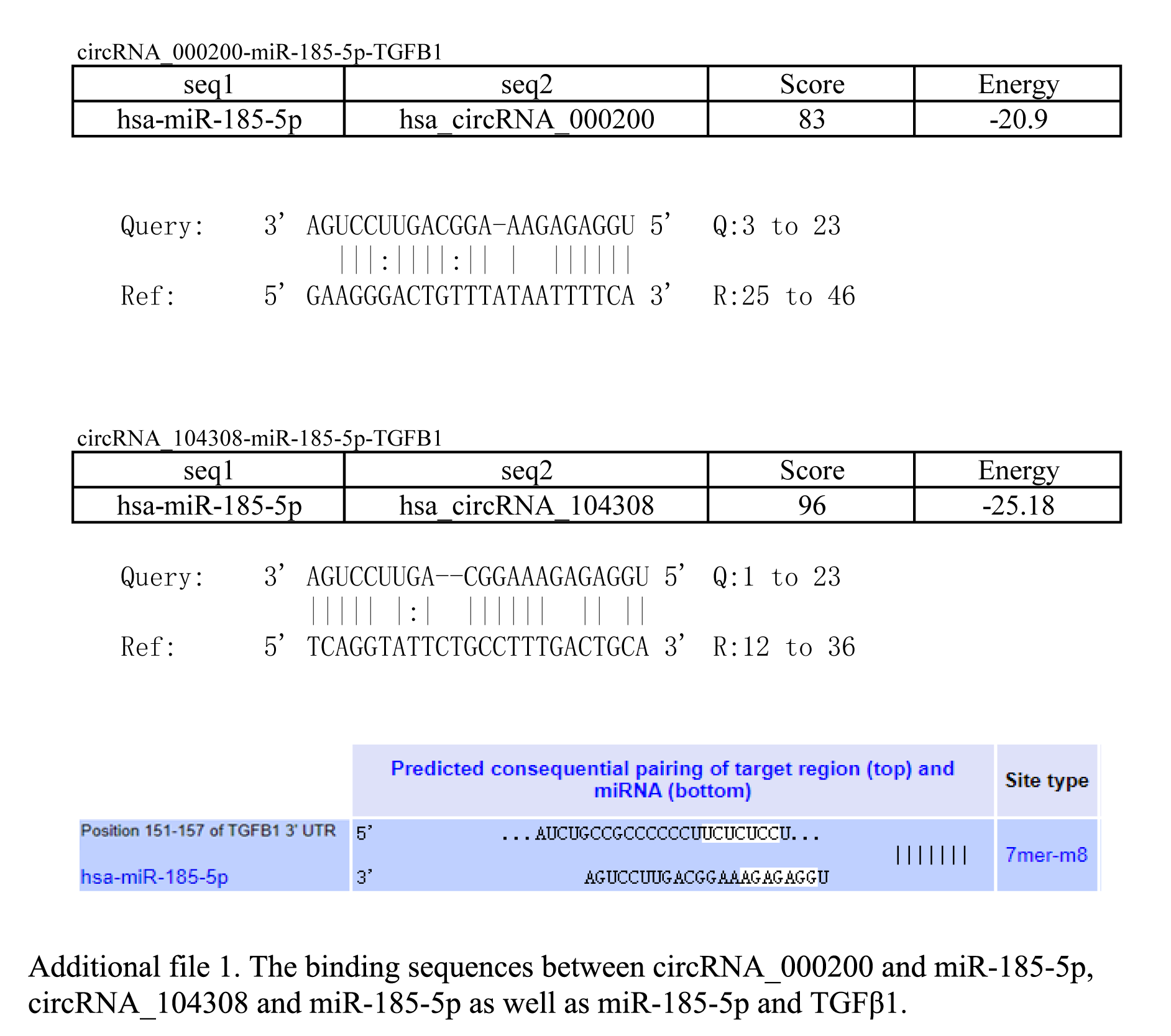

Supplement: Supplementary file 1 — Additional file 1. The binding sequences analysis of circRNA_000200/ circRNA_104308-miR-185-5p-TGFß1. [file 12891_2022_5579_MOESM1_ESM.tif]

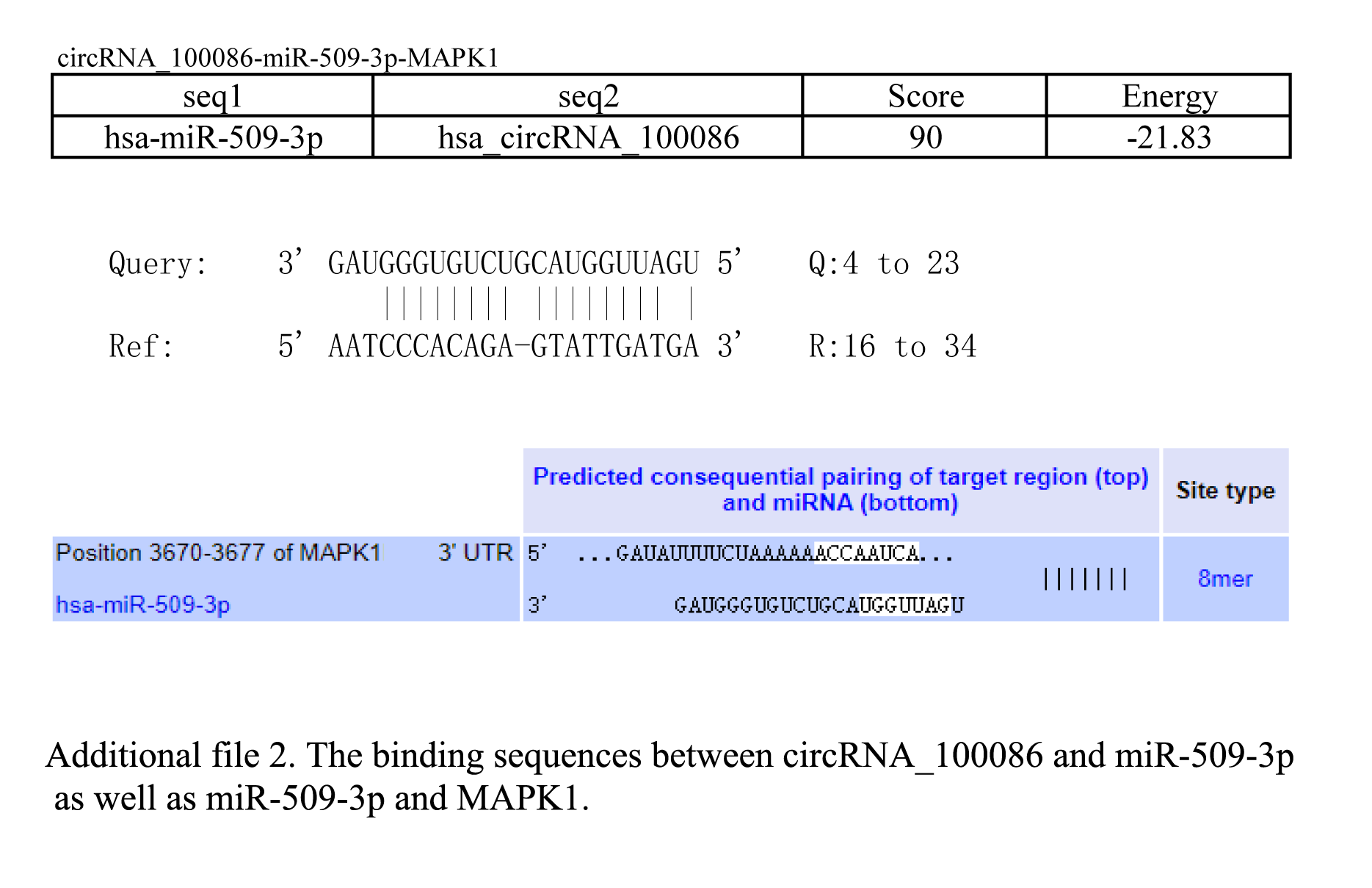

Supplement: Supplementary file 2 — Additional file 2. The binding sequences analysis of circRNA_100086-miR-509-3p-MAPK1. [file 12891_2022_5579_MOESM2_ESM.tif]

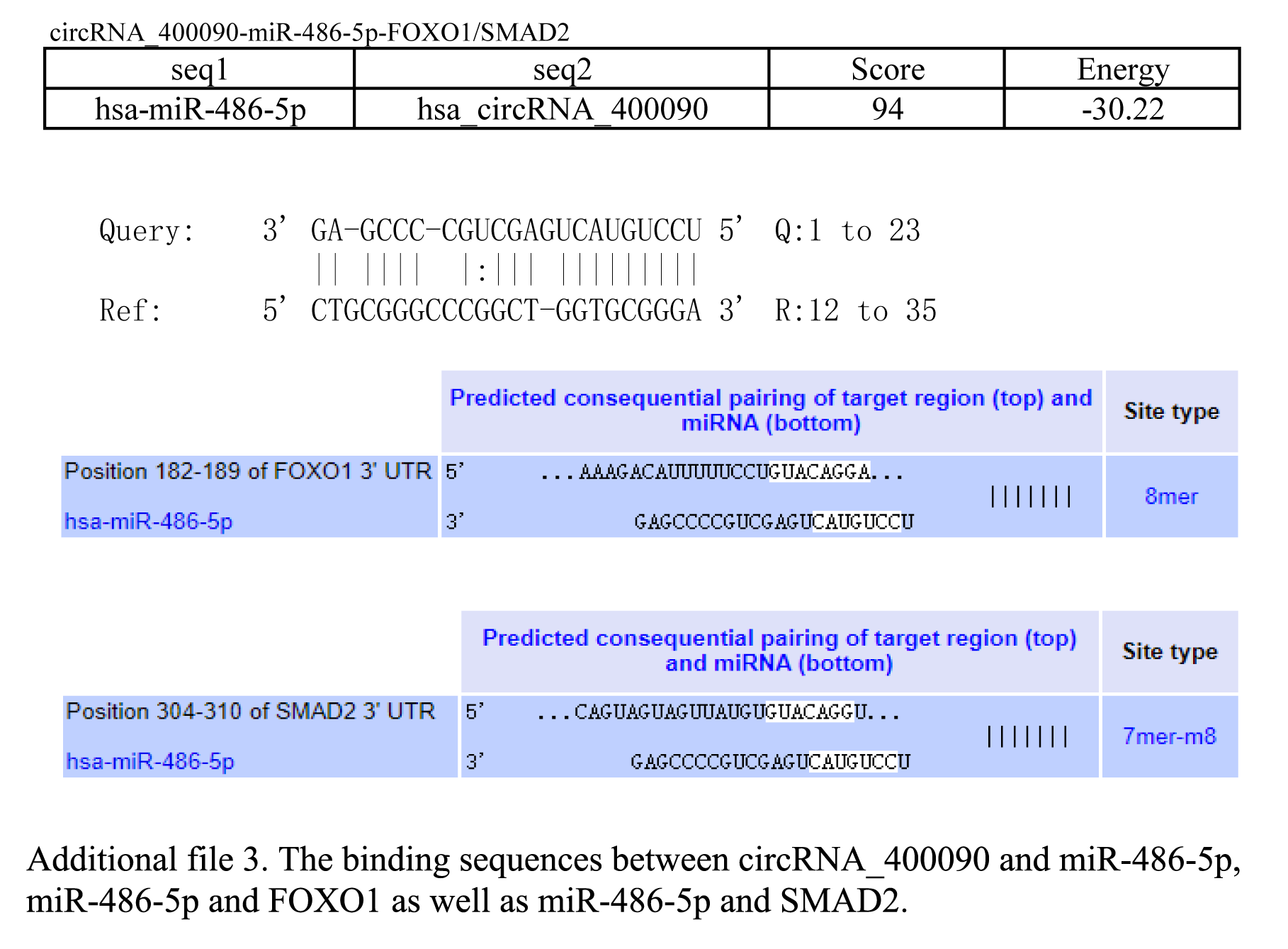

Supplement: Supplementary file 3 — Additional file 3. The binding sequences analysis of circRNA_400090-miR-486-5p-FOXO1/SMAD2. [file 12891_2022_5579_MOESM3_ESM.tif]
